# Older Consumers’ Readiness to Accept Alternative, More Sustainable Protein Sources in the European Union

**DOI:** 10.3390/nu11081904

**Published:** 2019-08-15

**Authors:** Alessandra C. Grasso, Yung Hung, Margreet R. Olthof, Wim Verbeke, Ingeborg A. Brouwer

**Affiliations:** 1 Department of Health Sciences, Faculty of Science, Vrije Universiteit Amsterdam, Amsterdam Public Health Research Institute, De Boelelaan 1085, 1081 HV Amsterdam, The Netherlands; 2 Department of Agricultural Economics, Ghent University, Coupure links 653, B-9000 Ghent, Belgium

**Keywords:** community-dwelling older adults, consumer, dietary protein, protein-energy malnutrition (PEM), sustainability

## Abstract

Protein-energy malnutrition (PEM) is a growing concern on account of an aging population and its negative health consequences. While dietary protein plays a key role in the prevention of PEM, it also plays a pivotal role in the environmental impact of the human diet. In search for sustainable dietary strategies to increase protein intake in older adults, this study investigated the readiness of older adults to accept the consumption of the following alternative, more sustainable protein sources: plant-based protein, insects, single-cell protein, and in vitro meat. Using ordinal logistic regression modeling, the associations of different food-related attitudes and behavior and sociodemographics with older adults’ acceptance to consume such protein sources were assessed. Results were obtained through a consumer survey among 1825 community-dwelling older adults aged 65 years or above in five EU countries (United Kingdom, the Netherlands, Poland, Spain, and Finland). Dairy-based protein was generally the most accepted protein source in food products (75% of the respondents found its consumption acceptable or very acceptable). Plant-based protein was the most accepted alternative, more sustainable protein source (58%) followed by single-cell protein (20%), insect-based protein (9%), and in vitro meat-based protein (6%). We found that food fussiness is a barrier to acceptance, whereas green eating behavior and higher educational attainment are facilitators to older adults’ acceptance to eat protein from alternative, more sustainable sources. Health, sensory appeal, and price as food choice motives, as well as gender and country of residence were found to influence acceptance, although not consistently across all the protein sources. Findings suggest that there is a window of opportunity to increase older adults’ acceptance of alternative, more sustainable protein sources and in turn increase protein intake in an environmentally sustainable way in EU older adults.

## 1. Introduction

The world’s population is estimated to reach 9.8 billion by 2050, and the number of persons aged 60 or above is expected to double compared to 2017 [1]. In the European Union (EU), one in five persons is already aged 65 or above, with the majority residing independently at home [2,3]. Protein-energy malnutrition (PEM) is a common and often underdiagnosed condition in this population and has serious consequences for health, functioning, and quality of life [4,5]. The risk of PEM in older adults is high because age-related changes in physiological, psychological, and environmental factors can disrupt the balance between dietary consumption and nutritional requirements [6]. The need for adequate consumption of protein, in particular, is increasingly recognized to prevent PEM and to enhance healthy aging [7,8]. It is argued that the recommended intake of protein inadequately meets the actual protein requirement of older adults and thus needs to increase to support good health and prevent decline in functional status in this population [6,9]. 

The challenge of meeting the higher protein requirement of an expanding population is compounded by environmental challenges, including climate change, biodiversity loss, land use change, and freshwater use [10]. Protein production, and food production in general, have a large impact on the environment, with animal-based protein production having a greater impact than plant-based protein production [11,12]. Relative to plant-based protein sources, animal-based protein sources are associated with more greenhouse gas emissions (GHGE) [13], greater requirements of land and nitrogen [14], and a greater impact on terrestrial and aquatic biodiversity [10]. Animal-based protein accounts for the majority (55% to 73%) of total protein consumed in the EU diet, with the contribution of protein derived from meat, dairy, eggs, and fish varying between countries [15]. A smaller percentage (24% to 39%) of total protein intake comes from plant origin, with the largest contribution of protein being derived from cereals [15]. The current ratio between animal and plant protein threatens the environment to the extent that business-as-usual for consumption and production is no longer an option [10,16]. 

In search of more sustainable protein sources, researchers are investigating the nutritional and environmental profiles of alternative protein sources that can act as meat substitutes, such as plant-based sources, insects, single-cell protein (e.g., mycoprotein such as Quorn^TM^ or microalgae), and in vitro meat (also known as lab-grown, cultured or clean meat) [17]. Indeed, such alternative protein sources have been found to have environmental benefits and be comparable to animal-based protein sources in terms of protein content [18,19]. Some alternative sources even outperform animal-based protein sources with regard to nutritional content [18]. The protein quality of such alternative protein sources, however, is slightly lower than of animal-based sources, although when eaten in a diverse diet, the quality of the total daily protein intake remains high [20]. Until recently, foods containing plant-based protein were a niche market targeting vegetarian and vegan consumers, but now they are consumed by a wider range of consumers who for various reasons want to reduce their meat intake [21]. Other alternative protein sources remain a niche market but are expected to shift to a wider market in the next decade [17]. Single-cell proteins are well-established in the market, yet are limited to Quorn^TM^ and algae-derived food supplements [22]. Edible insects are produced and sold in only a few EU countries, including the United Kingdom (UK), the Netherlands, and Finland, but are not yet widely available due to safety concerns [23]. In vitro meat has not yet penetrated the market but is expected to become commercially available in the coming years [24]. 

Considerable efforts have been made in recent years to determine opportunities and barriers for consumers to reduce their meat intake and to consume such alternative protein sources. Increasing evidence indicates that there is low consumer awareness of the environmental impact of meat production, as well as low willingness to change meat consumption behavior in terms of reducing or substituting meat in Europe [25]. Despite a seemingly close match between consumers’ image of a sustainable, a healthy, and a plant-based diet [26], it appears that very few consumers are willing to change meat consumption behaviors for sustainability reasons [25]. Barriers to changing meat consumption behaviors include preconceptions towards vegetarian diets, habits and prices, lack of familiarity with meat substitutes, and lack of skills to prepare meals containing meat substitutes [27,28]. In addition, food neophobia—the aversion of unfamiliar foods—and the food choice motive sensory appeal have been identified as barriers to consumer acceptance of meat substitutes [29]. Food choice motives related to health and environmental impact, however, have been shown to play a facilitating role in changing meat consumption behaviors [30]. Moreover, carrying out sustainable food-related activities such as buying local or organic food, which has previously been defined by Weller et al. as green eating behavior, has been correlated with lower meat intake [31]. Other factors that have been found to influence meat consumption behavior and alternative protein uptake are sociodemographics such as gender, age, and education [23,32,33]. 

Previous studies in this field consist mainly of younger adults, as they are the consumers of the future. Very little is known of the attitudes towards alternative, more sustainable protein sources among community-dwelling older adults in Europe. To sustainably meet the increased protein need in general, and of older adults in particular, it is imperative that the growing population accepts plant-based and other alternative proteins in favor to animal-based protein. Food choices of older adults are therefore crucial in the transition towards more sustainable diets. 

The objective of this study was to investigate consumer readiness to accept alternative, more sustainable protein sources among community-dwelling older adults (aged 65 years or above) in the EU. First, this study assessed the level of acceptance to consume the following alternative, potentially more sustainable protein sources: plant-based protein, insects, single-cell protein, and in vitro meat. Second, we investigated how different food-related attitudes and behavior (i.e., food fussiness, food choice motives, and green eating behavior) and sociodemographics (i.e., age, gender, country of residence, education) influence the acceptance to consume such protein sources. Insight into the readiness of older adults to accept alternative, more sustainable protein sources will help researchers and the food industry in particular to develop products that specifically target the preferences of older consumers with the overall aim to increase dietary protein intake within this population in an environmentally sustainable manner. 

## 2. Materials and Methods 

### 2.1. Study Design and Sampling

The present study was conducted within the PROMISS (PRevention Of Malnutrition In Senior Subjects in the EU) project, a five-year multicountry project funded by the European Commission (EC) Horizon 2020 aiming to understand the relationship between food, physical activity and biological changes and to develop dietary and physical activity strategies for the prevention of PEM among older European adults. This study used cross-sectional quantitative survey data that were collected electronically in June 2017 in five EU countries, namely, the United Kingdom, the Netherlands, Poland, Spain, and Finland; n = ±365/country.

Participants (n = 1825) were recruited by a professional market research agency using probabilistic sampling from an online access proprietary panel. Recruitment criteria and quota were established for older adults (65 years or above) who live independently. A nationally representative sample was achieved with additional measures performed by the market research agency, in terms of gender and region in each of the study countries, following a standard procedure: 1. The selection of potential participants was based on the background information collected during the registration survey, profiling, and screening surveys as performed by the recruitment agency; 2. specified quotas were established for gender (an equal amount of female and male) and regions proportional to the distribution within the overall population; and 3. the panelists were invited at various and designated times with close monitoring of participation and eventual corrective action during the fieldwork to ensure that the quotas for gender and region were fulfilled correctly. The same market research agency was responsible for all recruitment and contact procedures and electronic questionnaire administration. Ethics approval for the study was granted by the Belgian Ethics Committee of Ghent University Hospital in March 2017 (Reference No. B670201422567). 

### 2.2. Questionnaire and Scales

The development of the questionnaire regarding translation and pretesting has been elaborated in Hung et al. [34]. The questionnaire started with a short description of the PROMISS project and an informed consent and was followed by a screening for sample selection based on gender, age, region, and current living condition. The questions consisted of various sections including dietary habits, food-related attitudes and behaviors, acceptance towards various protein sources, sociodemographics and personal information. Order bias was avoided by rotating items within a question. 

#### 2.2.1. Dietary Habits

Regarding dietary habits, respondents were asked to indicate the frequency of consumption of various protein-rich food products, such as legumes, cooked meat, and cheese, while considering the last four weeks as the reference period. Furthermore, they were asked if they were currently following any dietary regime, such as a vegetarian diet that includes eggs and/or dairy products, a vegan diet, or any other diet regime, which they could specify. Respondents who reported following a vegetarian or vegan diet or reported having eaten meat (meat in a warm meal or cold-cuts) once a week or less in the past four weeks were considered to follow a meat-limiting diet.

#### 2.2.2. Food Fussiness

A food fussiness scale was adapted from den Uijl et al. [35] and Wardle et al. [36] to assess the degree to which one is selective about the range of foods that are accepted. The food fussiness scale consisted of seven items, e.g., ‘I enjoy tasting new foods’, in which respondents could indicate their level of agreement on a five-point scale from ‘Strongly disagree’ (=1) to ‘Strongly agree’ (=5). 

#### 2.2.3. Food Choice Motives

Food choice motives were assessed using a modified food choice questionnaire (FCQ) based on the scale developed by Steptoe and colleagues [37]. The modified FCQ consisted of 23 items and five factors: health, convenience, sensory appeal, price, and sustainability factors. The health, convenience, sensory appeal, and price factors have been validated and used previously [38]. Each item was answered on a five-point scale ranging from ‘Not at all important’ (=1) to ‘Extremely important’ (=5).

#### 2.2.4. Green Eating Behavior

Green eating behavior was assessed using a modified scale based on Weller and colleagues [31], which provides insights related to environmentally-conscious eating. The scale consisted of five items in which respondents could indicate how often they consumed green food products on a five-point scale ranging from ‘Never’ (=1) to ‘Always’ (=5) or ‘I don’t know’. Green eating behavior included eating locally grown or produced foods, foods purchased directly from a farmer’s market, organic foods, foods with an environmental sustainability label (e.g., Rainforest Alliance), and foods with an ethical sustainability label (e.g., Fair Trade). 

#### 2.2.5. Acceptance to Consume Various Protein Sources

Acceptance to consume food products containing various dietary protein sources was assessed. Participants were asked to indicate their acceptance to consume food products containing the following seven protein sources: (1) plant-based protein (derived from soy, pea, rice, canola, etc.), (2) meat-based protein (derived from cattle, pigs, poultry, etc.), (3) dairy-based protein (derived from milk, cheese, etc.), (4) seafood-based protein (derived from fish, shrimp, etc.), (5) insect-based protein (derived from mealworms, crickets, etc.), (6) single-cell protein (derived from microorganisms like algae, yeast, fungi, bacteria), and (7) in vitro meat-based protein (lab-made or cultured meat). Respondents could indicate their acceptance on a five-point scale ranging from ‘Very unacceptable’ (=1) to ‘Very acceptable’ (=5) or ‘I don’t know’. In this report, plant-, insect-, single-cell-, and in vitro meat-based protein sources are considered to be alternative, more sustainable protein sources as they in general are expected to have a lower environmental impact compared to animal-based protein sources [18]. 

#### 2.2.6. Sociodemographics and Personal Information

The personal characteristics of respondents were assessed using a series of 17 questions. These included sociodemographics such as educational level, being the main household (HH) grocery shopper, HH income, food expenses, lifestyle such as smoking and alcohol use, and presence of various health problems. Monthly HH income was asked in euros (€) for the Netherlands, Finland, Spain, in pounds for the UK, and in złoty in Poland. For the purpose of this study, HH income categories (low, middle, high) were created based on country-specific distributions (converted to euros where applicable): for the UK, the Netherlands, and Spain, Low = less than €1499, Middle = €1500 to €2499, High = €2500 or more; for Finland, Low = less than €1999, Middle = €2000 to €2999, High = €3000 or more; and for Poland, Low = less than €500, Middle = €500 to €999, High = €1000 or more. The health status was assessed by asking respondents if they had any of the following 17 health problems: pain in mouth, teeth or gums; dry mouth; difficulty swallowing; difficulty chewing; overweight/obesity; underweight; cardiovascular/heart disease; hypertension (high blood pressure); irritable bowel syndrome; other digestive problems; diabetes or high blood sugar levels; high blood cholesterol levels; cancer; food allergy; food intolerance; chronic kidney disease; and other chronic diseases or pain in general.

### 2.3. Statistical Analysis 

Descriptive statistics were used to report frequency and percentages for categorical variables and means and standard deviations for continuous variables. Level of acceptance to eat alternative, more sustainable protein sources, as well as other dietary protein sources, was reported as percentages. Differences between background characteristics of those who answered ‘I don’t know’ when asked to what extent they accept eating foods that contain an alternative, more sustainable protein source and those whose answer was within the range of the ordered scale for the alternative, more sustainable protein sources (very unacceptable (=1) to very acceptable (=5)) were tested using a Chi-square test or a t-test per protein source.

Exploratory factor analysis using principal axis factor analysis with varimax rotation, a type of orthogonal factor rotation, was conducted to check construct unidimensionality of food choice motives, food fussiness, and green eating behavior. Principal axis factoring finds the common variance among the items and identifies the factors or dimensions underlying the data [39]. Factors were considered reliable when Cronbach’s alpha internal reliability coefficient was above the lower limit of 0.6 [39]. 

Ordinal logistic regression analyses were conducted to identify determinants influencing older adults’ acceptance to eat sustainable protein sources, namely plant-, insect-, single-cell-, and in vitro meat-based protein. Those who responded ‘I don’t know’ when asked to what extent they accept eating foods that contain an alternative, more sustainable protein source were excluded from the regression analyses because this response option does not fit in the ordered scale. In total, 7%–11% respondents were excluded. Furthermore, because of the very small proportion of respondents reporting finding the consumption of insect- (1.6%), single-cell- (3.5%), and in vitro meat-based (0.9%) protein sources to be ‘very acceptable’ and plant-based protein sources (4.4%) to be ‘very unacceptable’, the ordinal scale of the alternative sustainable protein sources was collapsed to an ordered scale of three levels: unacceptable, neutral, and acceptable. Separate ordered logit models were tested with each alternative sustainable protein source as a dependent variable (ordinal) and sociodemographics (i.e., gender (nominal), age group (nominal), country of residence (nominal), education (nominal)), food-related attitudes (i.e., food choice motives (interval), food fussiness (interval)), and green eating behavior (interval) as determinants.

Assumptions for ordinal logistic regression were tested and validated. Correlation matrices of all the potential explanatory variables were examined to check for multicollinearity prior to conducting the regression analyses. We considered multicollinearity if the correlation coefficient between two explanatory variables was larger than 0.8 [39]. There were no indications of multicollinearity (Appendix A Table A1). The parallel lines assumption for ordinal regression was met for all models (plant-based protein *p*-value = 0.079; insect-based protein *p*-value = 0.023; single-cell protein *p*-value = 0.023; in vitro meat-based protein *p*-value = 0.086).

Statistical significance was considered at the α level of 0.05. Data were analyzed using SPSS version 24.0 (IBM Corp. Released 2016. IBM SPSS Statistics for Windows, Version 24.0. Armonk, NY, USA: IBM Corp.). 

## 3. Results

### 3.1. Characteristics of the Sample 

Background characteristics of the respondents are shown in Table 1. The sample of older adults aged 65 years and older was equally represented by gender and country. It had a higher share of lower-educated respondents and respondents living at home with others and responsible for most of the food shopping. On average, the respondents reported having 2 out of 17 asked health problems, with the most reported health problems being hypertension (42%), overweight/obesity (37%), and high blood cholesterol levels (29%). By far the majority (86.8%) of the respondents did not follow a meat-limiting diet. 

### 3.2. Factor Analysis 

The principal axis factor analysis confirmed the unidimensionality of the constructs of health convenience, sensory, price and sustainability food choice motives, food fussiness, and green eating behavior (Appendix A Table A2). All factors had sufficient internal reliability (health food choice motive α = 0.90; convenience food choice motive α = 0.85; sensory food choice motive α = 0.86; price food choice motive α = 0.81; sustainability food choice motive α = 0.82; food fussiness α = 0.81; green eating behavior α = 0.81).

### 3.3. Acceptance Towards Different Protein Sources 

**Figure 1:**
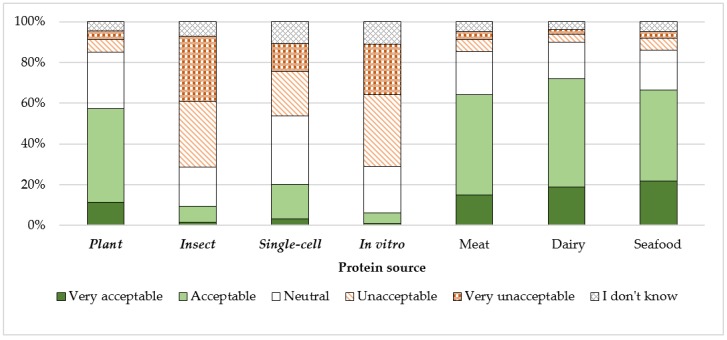
Level of acceptance to eat food products containing alternative, more sustainable protein sources (italicized and in bold) and other dietary protein sources in adults aged 65 years and older from five EU countries (%, n = 1825).

Figure 1 illustrates the level of acceptance to eat protein from various sources among the 1825 adults aged 65 years and older from five EU countries. In general, dairy-, seafood-, and meat-based protein sources were the most accepted protein sources, followed by plant-, single-cell-, insect-, and in vitro meat-based protein sources. Plant-based protein was the most accepted alternative, more sustainable protein source, with 46% of the respondents finding its consumption acceptable and 12% very acceptable. Next was single-cell-based protein, with 17% of the respondents finding its consumption acceptable and 3% very acceptable. Insect- and in vitro meat-based protein were the least accepted, with only 9% of the respondents finding the consumption of insect-based protein acceptable or very acceptable and 6% for in vitro meat-based protein. The percentage of respondents who reported ‘I don’t know’ was highest for single-cell- and in vitro meat-based protein (both 11%) compared to 7% for insect-based protein and 5% for plant-based protein. A greater proportion of respondents that responded ‘I don’t know’ to plant- and single-cell-based protein had lower educational attainment compared to those who responded using the ordered scale (X^2^ = 13.1, *p <* 0.001 for plant-based protein and X^2^ = 6.7, *p =* 0.010 for single-cell-based protein) (see Appendix A Table A3).

### 3.4. Determinants of Older Adults’ Acceptance to Eat Food Products Containing Alternative, More Sustainable Protein Sources

Table 2 shows that educational attainment, food fussiness, and green eating behavior are the most important factors influencing the acceptance to eat protein from alternative, more sustainable sources in adults 65 years and older from five EU countries. Higher-educated adults are 33%–41% more likely to accept eating food products containing alternative, more sustainable protein sources compared to those who did not complete higher education (*p* < 0.05). Similarly, a one-unit increase in green eating behavior is associated with an average 38% increase in the odds of being likely to accept alternative, more sustainable protein sources. Respondents who reported favoring more green eating behaviors are 29% more likely (95% CI 7%–57% more likely; *p* = 0.01) to accept in vitro meat-based protein sources, 36% more likely (95% CI 11%–66% more likely; *p* < 0.001) to accept single-cell-based protein sources, 44% more likely (95% CI 21%–72% more likely; *p* = 0.003) to accept insect-based protein sources, and 45% more likely (95% CI 21%–73% more likely; *p* < 0.001) to accept plant-based protein sources. 

By contrast, a one-unit increase in food fussiness score (i.e., those who are more selective about which foods they are willing to eat) is associated with an average 43% decrease in the odds of being likely to accept alternative, more sustainable protein sources. This ranges from a 27% decrease (95% CI 11%–40% decrease; *p* = 0.002) in the odds of being likely to accept in vitro meat-based protein to a 53% decrease (95% CI 44%–61% decrease; *p* < 0.001) in the odds of being likely to accept single-cell-based protein. 

The other studied determinants resulted in less consistent associations across all alternative, more sustainable protein sources. Gender was found to influence older adults’ acceptance towards the consumption of insect-, single-cell-, and in vitro meat-based protein sources, but not towards the consumption of plant-based protein sources. Female older adults are 57% less likely (95% CI: 45%–66% less likely; *p* < 0.001) to accept eating insect-based protein sources compared to male older adults. Furthermore, they were found to be 32% less likely (95% CI: 17%–45% less likely; *p* < 0.001) to accept eating single-cell based protein sources and 43% less likely (95% CI: 28%–55% less likely; *p* < 0.001) to accept eating in vitro meat-based protein sources. The country of residence also influenced older adults’ acceptability to eat sustainable protein sources except for single-cell-based protein sources. Compared to those living in the UK, those living in Poland are 61% more likely (95% CI 9%–138% more likely; *p* = 0.016) to eat plant-based protein sources and 39% less likely (95% CI: 7%–60% less likely; *p* = 0.022) to eat in vitro meat-based protein sources. Those living in the Netherlands and Finland are more than twice as likely (*p* < 0.001) and in Spain 1.5 times as likely (*p* = 0.042) to eat insect-based protein sources compared to the UK. Being 70 years or older compared to 65 to 69 years did not influence older adults’ acceptance to consume alternative, more sustainable protein sources. 

Out of the five food choice motives, the sensory motive has a negative influence on acceptance of insect- and single-cell-based protein sources. Older adults who find sensory attributes (smell, texture, etc.) of food important when making food choices are 17% less likely (95% CI: 1%–30% less likely; *p* = 0.040) to accept eating single-cell-based protein sources and 30% less likely (95% CI: 15%–42% less likely; *p* < 0.001) to accept eating insect-based protein sources. Older adults are 47% more likely (95% CI 19%–82% more likely; *p* < 0.001) to accept eating plant-based protein sources if they value health when making food choices. Older adults who find price of food important when making food choices are 25% more likely (95% CI 6%–48% more likely; *p* = 0.009) to accept in vitro meat-based protein sources. Convenience and sustainability food choice motives were not found to be significant determinants. 

The studied determinants of acceptance to eat food products containing alternative, more sustainable protein sources accounted for about 6.7%–15.2% of the variance, as shown by the Nagelkerke pseudo *R*^2^ (Table 2). 

## 4. Discussion

This study examined the level of acceptance to eat protein from alternative, more sustainable sources and the potential determinants of acceptance in a sample of 1825 community-dwelling adults aged 65 years and older from five EU countries. While the willingness to consume sustainable protein sources has been explored in younger adults [25], research in older adults is lacking, yet highly relevant due to the challenge of fulfilling the high protein requirement of the expanding older population in an environmentally sustainable fashion [9,40]. As dietary strategies to increase protein intake among older adults are sought [7], it is important and relevant that older adults’ attitudes towards sustainable food choices be further investigated.

### 4.1. Older Adults’ Acceptance to Eat Food Products Containing Alternative, More Sustainable Protein Sources

In agreement with previous research in younger populations, this study showed that there is low acceptance to eat food products containing alternative, more sustainable protein sources (i.e., insect-, single-cell, in vitro meat-based) among older adults in Europe [41,42,43]. The acceptance to eat food containing plant-based protein sources was higher compared to the innovative and more technology-driven alternative protein sources, but slightly lower compared to the acceptance to eat meat-, dairy-, and seafood-based protein sources in our sample. Similarly, a study conducted primarily among middle-aged adults found that there was more willingness to consume plant-based meat substitutes compared to protein from insects, yet people were even more willing to consume hybrid meat, meat with lower environmental impact, organic meat, and sustainably farmed fish [28]. The high acceptance to eat meat, dairy, and seafood among our sample of older adults underscores the important status of animal-based protein in the habitual Western diet [44,45]. 

It is possible that in our study respondents may have answered the question “to what extent do you accept eating food products that contain [alternative protein source]” without having adequate knowledge of the environmental and health benefits of the protein source, or even of the protein source itself [33]. A Belgium study found that only 13% of their study participants made up of mainly students knew the concept of in vitro meat without being given prior information [46]. In this study, only 11% of the participants reported ‘I don’t know’ when asked to what extent they accept eating single-cell- and in vitro meat-based protein. Furthermore, although all protein sources were presented in a consistent manner in the questionnaire, we speculate that compared to the familiar meat-, dairy-, and seafood-based protein sources, respondents may have not easily grasped the concept of eating unfamiliar protein sources such as insect-, single-cell-, and in vitro meat-based protein sources. Experimental research has shown that presenting such protein alternatives in the context of a meal or as an ingredient positively influences consumers’ acceptance of various meat substitutes and insects [27,47]. For instance, a study conducted in the Netherlands found that pizza with processed insect protein was more acceptable than a salad with visible insects [27]. Presenting various protein sources as abstract concepts (e.g., to what extent do you accept eating food products that contain insect-based protein, e.g., derived from mealworms or crickets) rather than in context of a meal may have negatively influenced consumers’ acceptance of protein alternatives [25].

### 4.2. Factors Influencing Acceptance

#### 4.2.1. Food-Related Attitudes and Green Eating Behavior

Food fussiness emerged as an important determinant of acceptance in our sample. Respondents with a higher degree of food fussiness were less likely to accept eating sustainable protein sources. Previous research has focused more on the concept of food neophobia, a construct that overlaps with food fussiness but refers specifically to the aversion of unfamiliar foods [48], as a barrier to the acceptance of novel foods [49,50], including meat substitutes [43], insects [41,51] and in vitro meat [46]. As plant-, insect- and single-cell-derived meat substitutes are relatively new products on the market, and in vitro meat-based food products have not yet penetrated the market [17], it is logical that fussy eaters would be less likely to accept these protein sources compared to less fussy eaters. 

There was no food choice motive that had a consistent, significant association with the acceptability to eat all four alternative, more sustainable protein sources, suggesting that expectations towards alternative, more sustainable proteins vary per protein source. In agreement with past research, we found that health motives contributed to the acceptability of consuming plant-based protein, and sensory appeal was a barrier to the acceptability of consuming insect- and single-cell-based protein [43,52]. These findings suggest that there are expectations of health-promoting properties with plant-based protein and taste deficiencies with insect- and single-cell-based protein. Surprisingly, price food choice motive had a positive influence on acceptance to eat food products containing in vitro-based protein. We expected that price consciousness would have a negative influence, as it has been previously found as a barrier to sustainable food choices [53] and to the acceptance of in vitro meat among a Belgium sample [46]. Further, in vitro meat is expensive (about $25 per kilo), although innovations are advancing to soon bring it into the market at a competitive price [17,54]. One way to explain our finding is the high level of unfamiliarity with in vitro meat in our sample. Although only 11% of the respondents reported ‘I don’t know’, it can be that the majority did not know the cost of in vitro meat-based protein. Eventually, participants may have assumed a low price for this product owing to the opportunity of industry-scale low-cost mass production without the need to raise, transport, and slaughter animals. 

Another striking result is that the sustainability food choice motive did not play an integral, consistent role in shaping the acceptability to eat sustainable protein sources. Sustainability food choice motive was expected be associated with a higher odds of accepting alternative, more sustainable protein sources because it was found to positively influence sustainable food consumption in previous studies [33,55]. However, our finding is in accordance with earlier studies that indicated that environmental concern does not directly translate into sustainable food choices, as many people may be either unaware of the environmental impacts of meat and other food products [28,56], or unable/unwilling to act in line with their pro-environmental attitudes [57]. Yet, it is possible that the potential impact of sustainability food choice motive has been covered in this analysis because is it significantly associated with other determinants, notably the health motive. The correlation between health food choice motive and sustainability food choice motive was statistically significant (although below the cutoff for multicollinearity), which is in line with the perceived match between health and sustainability in food as reported in the study by Van Loo and colleagues [26]. While convenience has been found to be an important motive for food choices made by older adults [58], it was not found to influence the acceptance to eat various sustainable protein sources in our sample. 

Consistent with expectations, those who reported practicing more green eating behaviors were more accepting of eating sustainable protein sources. Niva and colleagues [59] found that older adults (aged 65–80 years) were more active in sustainable food consumption practices, such as buying local or organic food, compared to younger adults in Scandinavia, suggesting that there is willingness among this population to make food consumption more sustainable. 

#### 4.2.2. Role of Sociodemographics

In line with previous studies, we found that higher-educated consumers are more likely to accept eating alternative, more sustainable protein sources [60]. Earlier studies have also shown that education level is inversely related to meat consumption [61]. One can speculate that higher-educated adults are more aware of the health and/or environmental benefits of eating a primarily plant-based diet. This is corroborated by Van Loo and colleagues, who found that individuals involved in health and/or sustainable eating are more likely to be higher educated than those not involved [26]. Gender and country of residence influenced acceptance of only certain alternative, more sustainable protein sources. Females were less likely compared to males to accept eating alternative, more sustainable protein sources, although there is evidence that females are more willing than males to reduce meat consumption [33,56]. Females have also been previously found to be less likely to accept insects [41], but more likely to accept of meat substitutes compared to males [32]. The identified country differences are empirical findings that may eventually be due to the different food cultures and penetration of alternative, sustainable protein sources in the market [62]. 

### 4.3. Strengths and Limitations of the Study

The major strengths of this study include the use of a cross-European large-scale survey and the investigation of various determinants of acceptance to eat sustainable protein sources in older adults. Our study has a few limitations, which were also previously described in Hung et al. [34]. In short, these include potential selection bias and limited generalizability, as the sample was restricted to older adults with online access and a certain level of skills to access and complete the online survey and potential social desirability bias of the self-reported measures. Furthermore, although all protein sources were presented in a consistent manner, the question assessing the level of acceptance towards various protein sources may be susceptible to bias. Compared to animal-based protein sources, the alternative, more sustainable protein sources are less familiar, and thus, respondents may have not been able to conjure up ways in which such protein sources can fit into their accustomed meal formats and food combinations. Previous research suggests that presenting such protein sources out of context may result in an inaccurate portrayal of the consumers’ actual attitude towards sustainable food choices [25]. However, even when placed in context, the acceptance of insect-, single-cell-, and in vitro meat-based protein sources is expected to remain lower compared to plant- and animal-based protein sources due to factors such as sensory appeal and perceived naturalness [25]. 

### 4.4. Implications of Findings and Future Research

The low acceptance of alternative, more sustainable protein sources could be a barrier to the transition to environmentally-friendly diets adequate in protein in older adults. However, the relatively high acceptance to eat more familiar plant-based protein sources may provide a window of opportunity to increase protein intake in an environmentally sustainable way. Recommendations to increase consumption of plant-based protein sources will be widely accepted by older adults, although other barriers to increasing food consumption in general, such as poor appetite, may arise [34]. The negative relationship between food fussiness and acceptability to consume sustainable protein sources also provides an opportunity. High food fussiness scores associate with more aversion to unfamiliar food as four of the seven factors are related to novel foods [48]. Previous studies found that the acceptance of alternative, more sustainable protein sources such as cultured meat increased after informing health and/or environmental benefits of the protein source [46]. Therefore, targeted information provision to increase awareness and familiarity of alternative, more sustainable protein sources might have a positive impact on the readiness to accept consuming these products. Furthermore, repeated exposure to the food has been shown to increase acceptability of Quorn and tofu in a Dutch sample [63]. 

While significant determinants of acceptance to eat sustainable protein sources were found in this study, the results show that much of the variance of acceptance is left unexplained. Other possible determinants of acceptance to eat sustainable protein sources may include familiarity [63], social norms, awareness, perceived consumer effectiveness, and perceived availability of the product [57]. Future research investigating additional determinants of attitudes towards sustainable food choices in older adults can consider placing the sustainable food choice in context by providing pictures of meals or products with, or real products of, for example, insects or insect-based protein as an added ingredient. Furthermore, acceptance and positive attitude does not necessarily translate into favorable intentions and behavioral change [57], and thus, further investigation into the intention or willingness to consume sustainable protein sources among older adults is warranted. 

## 5. Conclusions

This study provides insight into the readiness of older adults to accept alternative, more sustainable protein sources in the EU. To the best of our knowledge, this report is the first to focus on older adults’ attitudes towards sustainable food choices. Findings suggest that there is a window of opportunity to increase older adults’ acceptance of alternative, more sustainable protein sources and, in turn, increase protein intake in an environmentally sustainable way in EU older adults. Based on the results of this study, protein recommendations should focus on increasing consumption of plant-based protein sources as an alternative, more sustainable protein source, as these products will be the most accepted by older adults. Furthermore, our results indicate that older adults’ acceptability to consume alternative, more sustainable protein sources is influenced by food fussiness, green eating behavior, educational attainment, different food motives, gender, and country of residence. To increase older adults’ acceptance towards more innovative and more technology-driven alternative protein sources, evidence points to the need of greater awareness and familiarity of these products among this population. More studies are needed to investigate older adults’ acceptance of alternative, more sustainable protein sources after being provided information and/or being exposed to the protein source in a meal context, as well as their intention or willingness to consume sustainable protein sources. 

## Figures and Tables

**Table 1:** Background characteristics of 1825 adults aged 65 years and older from five EU countries (n = 1825 unless indicated otherwise).

Characteristic	% of Sample
Gender	
Male	50.4
Female	49.6
Age group	
65–69	55.9
70–90	44.1
Country	
United Kingdom	20.0
The Netherlands	20.1
Poland	19.9
Spain	20.0
Finland	20.0
Educational attainment	
Below tertiary level	59.6
Tertiary level or above	40.4
Perceived financial situation (n = 1791)	
Have some or severe difficulties	16.4
Get by alright	38.3
Manage quite or very well	45.3
Living condition	
Lives alone	30.6
Lives with others	69.4
Responsibility for food purchases	
Does most of food shopping	70.3
Shared responsibility for food shopping	19.6
Does not shop for food	10.1
Number of health problems ^1^, mean ± sd (n = 1748)	2.3 ± 2.1
Dietary regime ^2^	
Follows a meat-limiting diet	13.2
Does not follow a meat-limiting diet	86.8

^1^ Sum of number of reported health problems out of 17 asked health problems. ^2^ Meat-limiting diet includes those who reported to follow a vegetarian or vegan diet or reported to eat meat (meat in a warm meal or cold-cuts) one time per week or less in the past four weeks.

**Table 2:** Determinants of acceptance to eat food products containing protein from alternative, more sustainable sources in adults aged 65 years and older from five EU countries—results from ordinal regression analyses ^1^.

	Plant n = 1518			Insect n = 1483			Single Cell n = 1426			In Vitro n = 1435		
	OR	(95% CI)	*p*-value	OR	(95% CI)	*p*-value	OR	(95% CI)	*p*-value	OR	(95% CI)	*p*-value
Gender												
Male (Ref)	-			-			-			-		
Female	0.96	(0.78–1.19)	0.719	**0.43**	(0.34–0.55)	< 0.001	**0.68**	(0.55–0.83)	<0.001	**0.57**	(0.45–0.72)	<0.001
Age (y)												
65–69 (Ref)	-			-			-			-		
70–90	1.08	(0.87–1.33)	0.499	1.06	(0.84–1.34)	0.605	0.93	(0.76–1.14)	0.501	1.06	(0.85–1.33)	0.587
Country												
United Kingdom (Ref)	-			-			-			-		
The Netherlands	1.02	(0.73–1.43)	0.907	**2.16**	(1.48–3.15)	< 0.001	0.89	(0.64–1.23)	0.477	1.23	(0.86–1.76)	0.249
Poland	**1.61**	(1.09–2.38)	0.016	0.87	(0.55–1.37)	0.554	0.83	(0.58–1.18)	0.299	**0.61**	(0.40–0.93)	0.022
Spain	1.11	(0.79–1.56)	0.553	**1.50**	(1.01–2.21)	0.042	1.32	(0.96–1.84)	0.092	0.95	(0.66–1.36)	0.771
Finland	0.98	(0.71–1.36)	0.894	**2.23**	(1.56–3.17)	< 0.001	0.73	(0.54–1.01)	0.054	1.14	(0.81–1.61)	0.446
Educational attainment ^2^												
No higher education (Ref)	-			-			-			-		
Higher education	**1.33**	(1.06–1.66)	0.013	**1.40**	(1.11–1.78)	0.005	**1.41**	(1.14–1.73)	0.001	1.34	(1.06–1.70)	0.013
Food choice motives ^3^												
Health	**1.47**	(1.19–1.82)	<0.001	0.80	(0.64–1.02)	0.071	1.08	(0.88–1.33)	0.442	0.88	(0.70–1.11)	0.293
Convenience	0.88	(0.75–1.03)	0.118	0.96	(0.81–1.15)	0.675	0.96	(0.82–1.12)	0.592	1.05	(0.88–1.24)	0.593
Sensory	1.01	(0.84–1.20)	0.953	**0.70**	(0.58–0.85)	< 0.001	**0.83**	(0.70–0.99)	0.040	0.83	(0.68–1.00)	0.050
Price	1.10	(0.94–1.28)	0.254	1.13	(0.95–1.35)	0.159	1.11	(0.96–1.29)	0.159	**1.25**	(1.06–1.48)	0.009
Sustainability	1.04	(0.86–1.25)	0.691	1.22	(0.99–1.50)	0.059	1.10	(0.92–1.31)	0.302	0.98	(0.81–1.20)	0.881
Food fussiness ^4^	**0.55**	(0.45–0.66)	<0.001	**0.51**	(0.41–0.63)	< 0.001	**0.47**	(0.39–0.56)	<0.001	**0.73**	(0.60–0.89)	<0.001
Green eating behavior ^5^	**1.45**	(1.21–1.73)	<0.001	**1.36**	(1.11–1.66)	0.003	**1.44**	(1.21–1.72)	<0.001	**1.29**	(1.07–1.57)	<0.001
Nagelkerke R square (%)	12.1			15.2			12.5			6.7		

^1^ Odds ratio (OR) is bold if statistically significant, *p*-value < 0.05. ^2^ No higher education includes no education up to higher secondary education; Higher education includes bachelor, master or doctoral education. ^3^ Each food choice motive is a continuous score from one to five, with a greater score indicating more importance is placed on the respective motive (e.g., health) when making food choices. ^4^ Food fussiness is a continuous score from one to five, with a greater score indicating a greater tendency to be a fussy or picky eater. ^5^ Green eating behavior is a continuous score from one to five, with a greater score indicating a greater tendency to practice sustainable food-related behaviors such as buying local or organic food.

## Appendix A

**Table A1:** Correlation matrix of determinants in ordinal regression analyses for acceptance to consume sustainable protein sources in older adults in five EU countries ^1^.

Independent Variables	×1	×2	×3	×4	×5	×6	×7	×8	×9	×10
×1: Gender ^2^	–									
x2: Age ^3^	−0.043	–								
×3: Education ^4^	−0.079 **	0.030	–							
×4: Health ^5^	0.158 **	−0.053 *	0.047 *	–						
×5: Convenience ^5^	0.109 **	−0.031	0.008	0.338 **	–					
×6: Sensory ^5^	0.185 **	−0.073 **	−0.036	0.462 **	0.295 **	–				
×7: Price ^5^	0.057 *	−0.078 **	−0.105 **	0.361 **	0.446 **	0.289 **	–			
×8: Sustainability ^5^	0.202 **	−0.081 **	0.051 *	0.655 **	0.374 **	0.423 **	0.279 **	–		
×9: Food fussiness	−0.059 *	0.005	−0.066 **	−0.118 **	0.199 **	−0.088 **	0.104 **	−0.019	–	
×10: Green eating behavior	0.130 **	−0.058 *	0.134 **	0.247 **	−0.028	0.141 **	−0.070 **	0.429 **	−0.177 **	–

^1^ Items with asterisk are statistically significant, * *p*-value < 0.05, ** *p*-value < 0.01. ^2^ Gender was coded 0 = male, 1 = female. ^3^ Age was coded 0 = below 70 years, 1 = 70 years and older. ^4^ Education was coded 0 = No higher education, 1 = Higher education. ^5^ Food choice motive.

**Table A2:** Factor loadings from principal axis factor analysis in of 1825 adults aged 65 years and older from five EU countries.

Factor	Health ^1^	Convenience ^1^	Sensory ^1^	Price ^1^	Sustainability ^1^	Food Fussiness	Green Eating Behavior
*It is important to me that the food I eat on a typical day…*							
… contains a lot of vitamins	0.74						
… keeps me healthy	0.79						
… is nutritious	0.76						
… is high in protein	0.68						
… is good for my skin, teeth, hair, nails, etc.	0.73						
… is high in fiber and roughage	0.70						
… is easy to prepare		0.82					
… can be cooked very simply		0.91					
… takes no time to prepare		0.77					
… can be bought in shops close to where I live		0.53					
… is easily available in shops and supermarkets		0.43					
… smells nice			0.72				
… looks nice			0.82				
… has a pleasant texture			0.79				
… has no small pieces that go between my teeth ^2^			–				
… is easy to chew and swallow ^2^			–				
… tastes good			0.59				
… is not expensive				0.80			
… is cheap				0.78			
… is good value for money				0.56			
… is sustainable					0.35		
… is environmentally-friendly					0.71		
… is organic					0.70		
*To what extent do you agree or disagree with the statement:*							
I enjoy tasting new foods						0.73	
I enjoy a wide variety of foods						0.63	
I am interested in tasting food that I have not tasted before						0.76	
I refuse new foods at first						0.65	
I decide that I don’t like food, even without tasting it						0.61	
I am difficult to please with meals						0.46	
I refuse changing my daily dietary pattern						0.48	
*How often do you consume…*							
Locally grown or produced foods							0.681
Foods purchased directly from farmer’s markets							0.636
Organic foods							0.756
Foods with environmental sustainability label							0.836
Foods with ethical sustainability label							0.827
N	1825	1825	1825	1825	1825	1825	1808
Mean of construct	3.6	3.1	3.6	3.2	3.1	2.3	2.8
Standard deviation of construct	0.7	0.8	0.7	0.8	0.9	0.6	0.8
Cronbach’s α	0.90	0.85	0.86	0.81	0.82	0.81	0.81

^1^ Food choice motive; ^2^ Items were removed as they did not have a significant loading on any factor.

**Table A3:** Sociodemographic characteristics of the sample who responded ‘I don’t know’ (DK) versus the sample who responded in the ordered scale (R) (very unacceptable (=1) to very acceptable (=5)) when asked to what extent they eat foods containing a sustainable protein source ^1^.

Sociodemographic Characteristic	Plant			Insect			Single Cell			In Vitro		
	DK	R	*p*-value ^2^	DK	R	*p*-value ^2^	DK	R	*p*-value ^2^	DK	R	*p*-value ^2^
n =	84	1741		126	1699		195	1630		199	1626	
Gender												
Male	46.4	50.6	0.455	49.2	50.2	0.779	46.2	50.9	0.208	50.3	50.4	0.962
Female	53.6	49.4		50.8	49.5		53.8	49.1		49.7	49.6	
Age (y)												
Below 70	48.8	56.3	0.177	47.6	56.6	0.051	49.7	56.7	0.065	53.3	56.3	0.420
70+	51.2	43.7		52.4	43.4		50.3	43.3		46.7	43.7	
Country			0.009			0.052			0.013			0.192
United Kingdom	27.4	19.6		23.0	19.8		19.5	20.1		19.6	20.0	
The Netherlands	13.1	20.4		11.1	20.7		16.4	20.5		17.1	20.4	
Poland	8.3	20.5		19.8	20.0		14.9	20.6		16.1	20.4	
Spain	23.8	19.8		19.0	20.1		20.5	19.9		22.1	19.7	
Finland	27.4	19.6		27.0	19.5		28.7	19.0		25.1	19.4	
Educational attainment												
Below tertiary level	78.6	58.7	<0.001	67.5	59.0	0.063	68.2	41.4	0.010	65.3	58.9	0.082
Tertiary level or above	21.4	41.3		32.5	41.0		31.8	58.6		34.7	41/1	
Perceived financial situation	(n = 78)	(n = 1713)	0.320	(n = 122)	(n = 1669)	0.059	(n = 190)	(n = 1601)	0.818	(n = 191)	(n = 1600)	0.595
Have some or severe difficulties	12.8	16.6		13.1	16.7		17.9	16.2		14.1	16.7	
Get by alright	46.2	37.9		48.4	37.6		36.8	38.5		40.8	38.0	
Manage quite or very well	41.0	45.5		38.5	45.8		45.3	45.3		45.0	45.3	
Living condition												
Lives alone	27.4	30.8	0.508	30.2	30.7	0.905	27.8	31.0	0.346	26.6	31.1	0.295
Lives with others	72.6	69.2		69.8	69.3		72.3	69.0		73.4	68.9	
Responsibility for food purchases			0.740			0.729			0.477			0.983
Does most of food shopping	66.7	70.5		67.5	70.5		66.7	70.7		69.8	70.4	
Shared responsibility	21.4	19.5		22.2	19.4		22.6	19.3		20.1	19.6	
Does not shop for food	11.9	10.0		10.3	10.1		10.8	10.0		10.1	10.1	
Number of health Problems ^3^, mean ± sd	(n = 76)	(n = 1672)	0.585	(n = 116)	(n = 1632)	0.575	(n = 183)	(n = 1565)	0.452	(n =1 88)	(n = 1560)	0.632
	2.4 ± 2.2	2.5 ± 2.1		2.4 ± 2.1	2.5 ± 2.1		2.4 ± 2.1	2.5 ± 2.1		2.5 ± 2.1	2.5 ± 2.1	
Dietary regime ^4^												
Follows meat-limiting diet	15.5	13.0	0.518	12.7	13.2	0.876	17.4	12.6	0.061	16.1	12.8	0.195
Does not Follow Meat-Limiting Diet	84.5	87.0		87.3	86.8		82.6	87.4		83.9	87.2	

^1^ Presented as percent of total unless otherwise indicated. ^2^ Result from a test for significant difference between the two groups, using chi-square test or Fisher’s exact test for categorical variables or a t-test for continuous variables. ^3^ Sum of number of reported health problems out of 17 asked health problems. ^4^ Meat-limiting diet includes those who reported to follow a vegetarian or vegan diet or reported to eat meat one time per week or less in the past four weeks.
